# Endocrine mechanisms, behavioral phenotypes and plasticity: known relationships and open questions

**DOI:** 10.1186/1742-9994-12-S1-S7

**Published:** 2015-08-24

**Authors:** Michaela Hau, Wolfgang Goymann

**Affiliations:** 1 Max Planck Institute for Ornithology, Eberhard-Gwinner-Str., D-82319 Seewiesen, Germany; 2 University of Konstanz, Department of Biology, Universitätsstraße 10, D-78464 Konstanz, Germany

**Keywords:** hormone, behavior, reaction norm, environmental gradient, quantitative relationship, corticosterone, testosterone

## Abstract

Behavior of wild vertebrate individuals can vary in response to environmental or social factors. Such within-individual behavioral variation is often mediated by hormonal mechanisms. Hormones also serve as a basis for among-individual variations in behavior including animal personalities and the degree of responsiveness to environmental and social stimuli. How do relationships between hormones and behavioral traits evolve to produce such behavioral diversity within and among individuals? Answering questions about evolutionary processes generating among-individual variation requires characterizing how specific hormones are related to variation in specific behavioral traits, whether observed hormonal variation is related to individual fitness and, whether hormonal traits are consistent (repeatable) aspects of an individual's phenotype. With respect to within-individual variation, we need to improve our insight into the nature of the quantitative relationships between hormones and the traits they regulate, which in turn will determine how they may mediate behavioral plasticity of individuals. To address these questions, we review the actions of two steroid hormones, corticosterone and testosterone, in mediating changes in vertebrate behavior, focusing primarily on birds. In the first part, we concentrate on among-individual variation and present examples for how variation in corticosterone concentrations can relate to behaviors such as exploration of novel environments and parental care. We then review studies on correlations between corticosterone variation and fitness, and on the repeatability over time of corticosterone concentrations. At the end of this section, we suggest that further progress in our understanding of evolutionary patterns in the hormonal regulation of behavior may require, as one major tool, reaction norm approaches to characterize hormonal phenotypes as well as their responses to environments.

In the second part, we discuss types of quantitative relationships between hormones and behavioral traits within individuals, using testosterone as an example. We review conceptual models for testosterone-behavior relationships and discuss the relevance of these models for within-individual plasticity in behavior. Next, we discuss approaches for testing the nature of quantitative relationships between testosterone and behavior, concluding that again reaction norm approaches might be a fruitful way forward.

We propose that an integration of new tools, especially of reaction norm approaches into the field of behavioral endocrinology will allow us to make significant progress in our understanding of the mechanisms, the functional implications and the evolution of hormone–behavior relationships that mediate variation both within and among individuals. This knowledge will be crucial in light of already ongoing habitat alterations due to global change, as it will allow us to evaluate the mechanisms as well as the capacity of wild populations to adjust hormonally-mediated behaviors to altered environmental conditions.

## Introduction

Animals display a fascinating array of behaviors, including sophisticated foraging techniques, spectacular courtship displays and energetically demanding parental behavior. Behaviors can vary considerably among individuals of one population, for example individuals from many species consistently tend to respond in either a ‘bold’ or a ‘shy’ manner to a challenging situation like a novel environment (for example, differing in exploration speed and willingness to take risks [1]). Qualitative and quantitative aspects of behavior can also vary considerably within an individual, often within minutes or hours. This short-term variation can be exemplified by the drastic behavioral switch that animals can show upon the sudden appearance of a predator, when non-essential behaviors are rapidly suppressed in favor of fight-or-flight responses [2].

The type and quantity of behavior that is displayed often reflects the context: it may not pay to express an elaborate courtship display without a potential mate being around to watch. And it may be outright dangerous to produce conspicuous displays if a predator is nearby [3]. Further, behaviors are also regulated according to environmental conditions. For example diurnal animals like most bird species are active primarily during daylight hours but are inactive at night [4]. Hence, behaviors need to be displayed in the appropriate social context, at the right time, and in the right internal state. How can the expression of behaviors be regulated to be appropriate for a given situation?

Hormones, being internal signals, are known to be potent mediators of behavioral changes in vertebrates. For example, the reproductive hormone testosterone promotes the expression of courtship behavior and aggression [5-7]. At the same time, testosterone has been suggested to inhibit parental behavior (summaries in [7,8]). In contrast, the related glucocorticoid hormones can regulate locomotor and foraging activity according to environmental conditions, as well as behaviors expressed during acute and chronic challenging conditions [9-11].

By being released into the blood stream, hormones are systemic signals that can reach all parts of the body and affect multiple tissues at the same time. Their potential for exerting pleiotropic effects make hormones particularly suited for regulating complex phenotypic changes that involve multiple traits (e.g. [5,6,12,13]). Consequently, endocrine mechanisms are involved in major transitions between life-history stages (e.g., puberty, reproduction, molt/pelage change, migration, sex change [14]), in establishing links among traits to create behavioral suites (behavioral syndromes, coping styles; [15]), and in life-history trade-offs [5,16]. Hormones also act as transducers of environmental information to regulate behavior of individuals, as their regulation is highly sensitive to both a biotic and biotic changes in the environment (e.g. [17]).

While much has been learned about how hormones regulate behaviors in the past couple of decades [18,19], many basic properties are still unknown. For example, while earlier research has focused on identifying general mechanisms of hormonal action among species or populations, the causes and functional implications of individual variation in hormone concentrations are still not well-understood [19-22]. It is also still under debate whether hormones can be viewed as heritable traits, and thus whether hormone-behavior relationships can exhibit an evolutionary response to selection [12,23-27]. On a mechanistic level, even though some of the molecular mechanisms of hormone actions are being increasingly elucidated, quantitative patterns of the relationship between hormones and behavior within individuals have remained unclear [28,29]. Significant progress in our understanding of these issues will be essential to fully understand the mechanisms by which hormones allow individuals and populations to adjust behaviors to immediate local circumstances, and how such mechanisms can evolve to enable adaptations to changing environments.

Below, we will first summarize some of the basic mechanisms of hormonal regulation of behavior, focusing on steroid hormones (Section 2). We will then discuss issues pertaining to among-individual variation in hormone levels, such as their relationship to behavioral phenotypes and fitness, the repeatability of hormone concentrations over time, and possible implications for evolutionary change (Section 3). We will then consider in detail the topic of quantitative relationships between hormones and behavior within individuals (Section 4) and possible consequences for behavioral flexibility. In light of our own research foci and the availability of a large body of existing literature, we will utilize primarily examples for the two steroid hormones corticosterone and testosterone, and from avian species to illustrate our review. Furthermore, even though some of the actions of corticosterone and testosterone can affect phenotypes in a more permanent way (organizational effects), in this review we will focus on transient, i.e. activational effects [30]. Finally, it is always important to keep in mind that not only hormones can affect behavior, but behavior can also feed back on hormone concentrations, for example during social interactions [31-34]. We will briefly touch upon this in section 4, but for reasons of brevity, will not further elaborate on this issue here. A detailed review on behavioral feedbacks on hormones can be found in [33,34]. We will end this review by suggesting that the inclusion of tools like reaction norms into the field of behavioral endocrinology will be important for making progress in our understanding of hormone-behavior relationships and their evolution.

## Mediation of behavior by corticosterone and testosterone

The two steroid hormones corticosterone and testosterone are primarily synthesized in specific glands, the gonads and adrenals, respectively [18]. However, other tissues can also produce these hormones, most notably the brain [35]. The production of steroid hormones is regulated via both internal and external stimuli (e.g., biological clocks, releasing hormones, photoperiod, social factors) and upon production steroid hormones are released into the blood stream. They are synthesized from cholesterol and a variety of enzymes are involved in their synthesis. Once released into the blood stream; they can freely pass cell membranes and bind to intracellular receptors, form dimers and bind to steroid-response elements on the DNA, thereby exerting transcriptional effects [18]. Some steroid hormones are known to have membrane receptors, which exert their effects through second messenger systems that provide for faster actions (within seconds or minutes) than the activation of intracellular receptors (within 30-60 mins [36,37]). It is important to keep in mind that even though blood-borne signals like hormones can reach all cells in the body, only cells that express specific receptors can respond to that signal, thus ensuring tissue specificity of hormonal actions. The interactions of steroid hormones with their receptor, and hence their effects on traits, can further be modulated by steroid-binding globulins in the plasma [38,39], and by a broad range of additional molecules that can change the ensuing receptor-mediated processes. Finally, enzymes can convert steroid hormones into other active hormones or into inactive compounds [18].

Corticosterone is a metabolic hormone in birds, amphibians, reptiles and some mammals [18]. Being a glucocorticoid hormone, it has a prominent role in the regulation of blood glucose levels, interacting with insulin and glucagon to fine-tune processes related to the availability of energy resources versus their storage. At baseline concentrations, corticosterone is responsible for many ‘house-keeping’ processes such as maintaining energy homeostasis within a certain range, but also for modulating metabolic processes on a diel basis [40,41]. For example, in most day-active animals, circulating corticosterone levels increase in late night or early morning to prepare the organism for daily activities [42-44]. Baseline concentrations of corticosterone are also known to rise during times of increased energy demand like when thermoregulation is increased in cold weather, during demanding parental phases and in times of elevated locomotor activity [40,45]. Corticosterone has a dual role, also rapidly increasing from baseline concentrations to stress-induced levels after an individual experiences a challenging stimulus [9,10,46]. Such stress-induced corticosterone concentrations promote behavioral changes that help cope with but also recover from an acute challenging experience, like increases in locomotor and foraging activity and decreases in non-essential behaviors like sexual activity [9,47,48]. Stress-induced concentrations of corticosterone typically peak within 30-60 minutes after the onset of the acute challenge, but subsequently are downregulated again to baseline concentrations by a process called negative feedback. This downregulation is important for allowing the individual to resume normal activities, and to avoid prolonged exposure to increased corticosterone levels, which can have adverse effects over long timespans [10].

The sex steroid testosterone is a key hormone with regard to sperm production, sexual behavior, the development of some secondary sexual characters or ornaments, and the expression of agonistic behaviors in a reproductive context (also including other morphological, physiological and behavioral traits; e.g. [6,7,28,49,50]). Testosterone is mainly produced by the Leydig cells of the male testis, but females can also have substantial levels of testosterone that can be of ovarian or adrenal origin. The brain of females and males can also be a source of testosterone or other sex steroids (reviewed by [35]). Testosterone can either act directly by binding to the androgen receptor, as a prohormone that is converted to dihydrotestosterone (DHT, which has an even higher affinity to the androgen receptor), or after local conversion to estradiol, which then can bind to an estrogen receptor (e.g., [18]). Testosterone has gained substantial interest in studies of evolutionary ecology, in particular because of its potential role in mediating trade-offs between reproduction and self-maintenance, or between mating and parental effort (e.g. [5,6]).

## Among-individual variation in hormone concentrations: relationships to behavioral phenotypes and fitness

**Figure 1:**
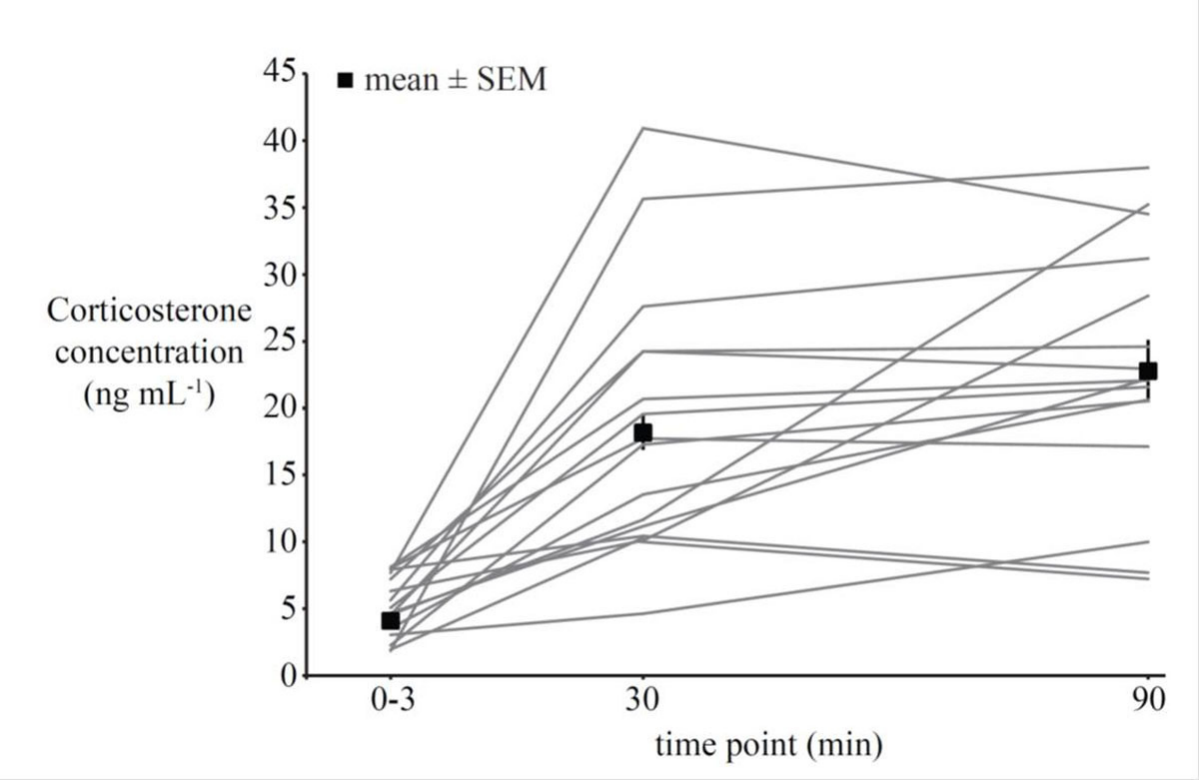
**Among-individual variation in corticosterone concentrations of wild great tits.** Circulating concentrations of corticosterone (ng/ml) were assessed within 0-3 minutes, and 30 and 90 minutes, respectively, of a capture-restraint protocol. Solid lines connect individual data points (n=16), solid squares indicate mean±SEM values (n=82 for 0-3 min, and 30 min, resp.; n=16 for 90 min). [51]

When we collect plasma samples from individuals of a population at a particular point in time, we typically encounter conspicuous variation in circulating hormone concentrations (Fig. 1) [20,21,51-55]. So why does, for example, at the same time of day, in the same habitat and population and with the same sampling procedure, one individual show two-or threefold higher concentrations of a particular hormone than its neighboring conspecific? Is this variation meaningful, i.e. is it related to an individual's behavioral phenotype and fitness, to environmental influences, or does it represent random variation (e.g., sampling error or measurement error or both)? Further, are hormone concentrations (and their effects on behavior) consistent for a given individual, and how heritable are hormonal traits? If variation in hormone concentrations was truly among individuals, was related to fitness, and was consistent for an individual there would be a potential for selection to act on this hormone-behavior-fitness relationship. Such basic questions in evolutionary endocrinology are still hardly understood, but essential for increasing our understanding of whether and how fast evolutionary change may occur in the endocrine mechanisms that enable behavioral adaptation to changes in the environment.

Below we will review some of the findings from our research, which aims at increasing our understanding of microevolutionary processes in hormonal (corticosterone) traits in a wild vertebrate species, the great tit (*Parus major*) [51,53,56-62]. While there are several research avenues by which questions in evolutionary endocrinology can be approached [12,26,63,64], thus far we have focused on characterizing natural variation in corticosterone concentrations in wild great tit populations, relating variation in hormone concentrations to behavioral phenotypes and fitness of individuals. Further, we will review studies (including our own) on individual consistency (repeatability) of corticosterone concentrations in great tits to discuss the potential for selection to act on individual phenotypic traits. The great tit research will then be evaluated in relation to studies on other species and hormones, to begin to shed light on the question of how hormone-behavior relationships may evolve*.*

### Does natural variation in hormone levels relate to behavioral phenotypes?

In a first set of studies, we tested whether corticosterone levels of great tits were related to individually consistent behavioral characteristics often referred to as ‘personality’ (e.g., [1,65,66]). We chose to study the personality trait of exploratory behavior, ranging on a continuum from a slow to a fast exploration speed of novel environments, because in great tits it has been shown to be highly repeatable, substantially heritable and under both natural and sexual selection [67-73]. In great tits, exploration speed is also correlated with the willingness to approach a novel object (‘boldness’), as well as with risk-taking, aggression and dominance [74,75]. Furthermore, bi-directional selection lines have been created for fast-bold versus slow-shy individuals [76]. Since tests to assess personality involve exposure to conditions that are likely intimidating (unfamiliar rooms or objects), we tested the prediction in adult great tits that, as in rodents ([15], but see [77]) fast-bold individuals should show lower increases in corticosterone concentrations following exposure to a standardized stressor compared to slow-shy individuals. As a standardized stressor, we employed a ‘capture-restraint protocol’ commonly used in avian field studies, for which a first blood is taken within 3 minutes after capture for baseline corticosterone, the bird is then being held in an opaque cotton bag and re-sampled after 30 minutes to assess stress-induced corticosterone concentrations [56,78].

**Figure 2:**
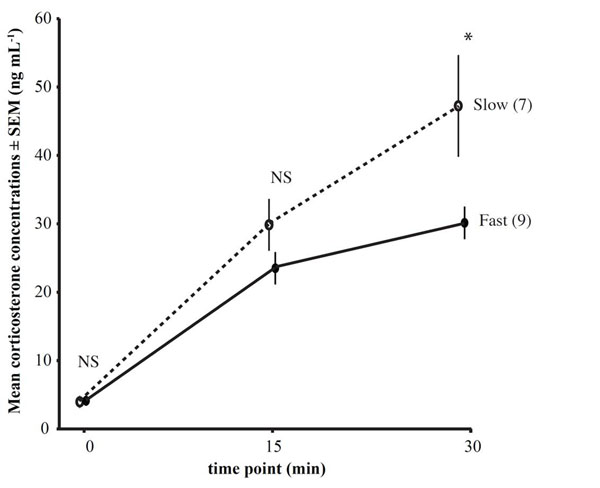
**Corticosterone concentrations during a capture-restraint protocol on captive great tits from a selection line.** Circulating baseline and stress-induced corticosterone concentrations of 4^th^ generation individuals from a selection line on fast-bold (filled symbols and solid line, n=9) and slow-shy (open symbols and dashed line, n=7) behavior sampled over time (mean±SEM; repeated measures general linear model: NS=non-significant; *p<0.05) [62].

**Figure 3:**
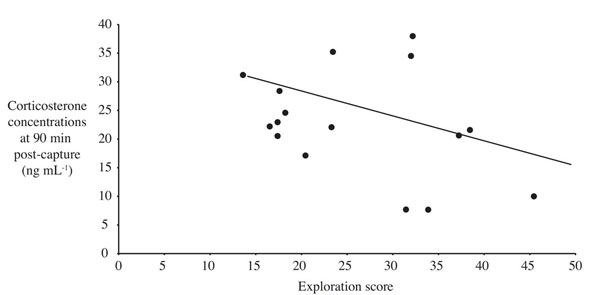
**Relationship between exploration speed and corticosterone concentrations in wild great tits.** Negative relationship between exploration score (higher numbers indicate a faster exploration of a novel room) and corticosterone concentrations 90 minutes after the onset of a capture-restraint protocol in wild great tits (n=16, partial correlation coefficient from general linear model: r=-0.57, p=0.032). Note that the data depicted here were obtained from the same individuals as those depicted with solid lines in Fig. 1 [51].

Confirming our predictions, great tits derived from the fourth generation of a selection line on fast exploratory behavior reached significantly lower stress-induced corticosterone concentrations within 30 minutes of the capture-restraint protocol compared to conspecifics selected for slow exploration speed (Fig. 2) [62]. There were no differences in concentrations of baseline corticosterone between the two groups. The trend for fast-bold individuals to show a lower glucocorticoid stress response than slow-shy individuals was further supported in a subsequent field study on great tits, which had been tested for their personality in captivity in the same standardized way as the selection line birds but subsequently released back into the wild. When tested for their corticosterone responses to the standardized stressor, wild individuals with a slow-shy personality increased corticosterone concentrations more rapidly within the first three minutes of the capture-restraint protocol, and still maintained higher corticosterone concentrations at 90 minutes (but not at 30 mins) after the onset of the capture-restraint protocol (Fig. 3 [51]). Taking the findings from captivity and the wild together, they support the notion of a faster, stronger and more protracted response of the endocrine stress axis to a standardized stressor in great tits with a slow-shy personality and a slower, lower and shorter response in fast-bold individuals.

In combination with earlier evidence [79,80], our results suggest that variation among great tit individuals in their corticosterone phenotype is linked with repeatable variation in a heritable behavioral phenotype that is fitness-relevant and under selection. These data fit with a general tendency among vertebrate species for proactive/bold individuals to have lower and reactive/shy individuals to have higher glucocorticoid stress responses ([15,81], but see [77,82]).

Natural variation in corticosterone levels in wild great tits also shows strong covariation with parental care, a behavioral trait that is repeatable in several avian species (e.g., [83,84]) and closely tied to reproductive success. Parental care, especially the number of trips to the nest to deliver food to the nestlings in great tits is a strong determinant of number of offspring produced during a breeding attempt [85]. Furthermore, in female blue tits (*Cyanistes caeruleus*) parental care is linked with exploratory and aggressive behavior, thus possibly providing a mechanistic link between personality and reproductive success [86]. In our study, baseline corticosterone concentrations were positively correlated with offspring provisioning rates in adults of both sexes: when individuals were sampled prior to the start of the breeding season their corticosterone concentrations predicted their subsequent parental provisioning rates, and there was also a relationship between baseline corticosterone and nestling care when birds were sampled during the parental phase in May [53], although in a different direction, see 3b]. Hence, among-individual variation in corticosterone concentrations was also related to a strongly fitness-relevant trait, parental behavior (the reasons for the existence of variation in parental provisioning rates will not be discussed here, but could relate to individual condition, plasticity and trade-offs; e.g., [84]). Why pre-breeding corticosterone concentrations predicted later nestling provisioning rates remains to be determined, but it could be related to individual quality or condition, i.e., the ability to perform demanding work. The relationship between baseline corticosterone and parental care during the breeding season likely is a causal one, as a slight experimental increase in baseline concentrations of corticosterone through slow-release implants during the breeding phase was effective in increasing reproductive investment in both males (increased feeding of their incubating female partner) and females (increased duration of incubation bouts of eggs and of brooding bouts of hatchlings [57]). During the breeding phase higher levels of corticosterone may function to mobilize energy for parental care; similar relationships have been shown in other species [54,59,87].

### Does natural variation in hormone levels relate to fitness?

**Figure 4:**
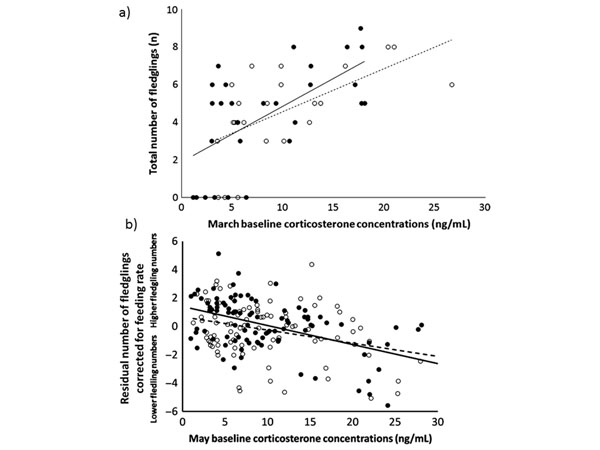
**Relationships between baseline corticosterone concentrations and fledgling numbers in wild great tits.** Baseline corticosterone concentrations (ng/ml) and yearly fledgling number (a) before the breeding season (March) and (b) during the peak parental provisioning phase in May (yearly fledgling number was corrected for feeding rate since fledgling number and feeding rate are positively correlated). Solid symbols and lines: females (March: n=26, May: n=96); open symbols and dashed lines: males (March: n=22, May: n=93), data from two study years are combined. Regression lines derived from linear mixed models, p<0.02 for all relationships [53].

Since among-individual variation in corticosterone in great tits was related to fitness-relevant behavioral phenotypes, it was logical to next predict that it will also directly predict fitness. Indeed, in a two-year field study on great tits, we found consistent linear relationships of baseline corticosterone levels and reproductive success, one major determinant of fitness (Fig. 4a,b) [53]. Interestingly, the relationship between baseline corticosterone levels and number of fledglings produced was positive for individuals sampled before the start of egg-laying in March (Fig. 4a). By contrast, this relationship was negative when individuals were sampled in the parental phase of the breeding season (Fig. 4b; we statistically controlled fledgling number for feeding rate, because it is one of major determinant of fledgling number, but this did not change the direction of the relationship with corticosterone). Thus, even though the relationship in the two seasonal stages was linear, its direction varied and there seemed to be seasonal variation in optimal hormonal phenotypes. Indeed, seasonally plastic individuals seem to fare best: those individuals that had the highest baseline concentrations before the breeding season in March, but when sampled again during the breeding season in May had the lowest levels produced the most offspring [53]. Hence, there is the possibility that certain corticosterone phenotypes of individuals (seasonally plastic in a certain direction) are selected for through fecundity selection.

Seasonal variations in the direction of the relationship between baseline corticosterone and reproductive success have also been observed in other songbird species [54,56]. In great tits, individuals with higher baseline corticosterone concentrations during the pre-breeding season may produce more offspring because they are able to invest more into reproductive processes, or because of increased thermoregulatory demands at this time of year in individuals with higher reproductive investment [53]. During breeding in May, individuals with lower baseline corticosterone concentrations fledged more offspring, possibly either because they were able to maintain lower baseline corticosterone levels despite a high parental effort (due to high quality and/or high-quality nesting habitat), or because they were paired with a mate showing high parental investment thus decreasing their own energetic demands and hormone levels [53]. More research is also needed to elucidate why at similar breeding stages there exists variation among species in the direction of the relationship between baseline corticosterone and reproductive success. In white-crowned sparrows (*Zonotrichia leucophrys oriantha*), like in great tits, there is a positive relationship between baseline corticosterone levels in the pre-breeding season and fecundity [88], while in house sparrows (*Passer domesticus*) the relationship is negative at the same seasonal stage [59]. It is tempting to speculate, but needs to be formally tested, that differences in the ecology of species, leading to divergent metabolic demands and patterns of reproductive investment explain these results. To this end, more longitudinal studies in wild populations involving the repeated sampling of individuals are urgently needed.

In our studies on great tits, we did not find relationships between variation in stress-induced corticosterone levels, and survival rates of individuals, unlike several previous studies on other species [88-92]. Instead, stress-induced corticosterone concentrations predominantly varied with environmental conditions, i.e., with weather and food abundance in a given season and year [53]. However, male great tits that reached higher stress-induced corticosterone concentrations during the capture-restraint protocol in the breeding season showed an increased likelihood to abandon their brood in a year with bad environmental conditions, and they abandoned their brood faster than males with lower stress-induced corticosterone levels [58]. Since great tits from our study population often are single-brooded, male nest abandonment can have severe fitness consequences, since in many cases females will consequently abandon the brood as well and the young will die. In this study, about half of the parents that had abandoned their first brood did re-nest. Interestingly, when raising their second broods and successfully fledging their offspring, these re-nesting males had lower stress-induced corticosterone concentrations than at the same stage during the first breeding attempt [58].

Finally, we discovered another, perhaps more indirect way in which corticosterone traits may be related to reproductive success: partners of a pair in which the similarity in corticosterone concentrations increased from the pre-breeding to the breeding season raised more offspring than pairs that did not become more similar in hormone concentrations [60]. Furthermore, pairs with more dissimilar baseline corticosterone levels were more likely to divorce after the breeding season and pair up with a new partner in the following year. These findings suggest a role not only for natural selection (see above), but also for sexual selection to shape corticosterone concentrations in wild great tits (for similar findings with testosterone, see [93]). However, more work is required to establish that selection is indeed acting on the genetic components underlying hormone concentrations and their regulation of behavioral traits versus a common environmental factor affecting both hormone-behavior relationship and fitness (e.g., [94,95]).

### Are hormone levels consistent within an individual?

Evolution can only happen if selection acts on the heritable component of a trait (see also above). Hence it is important to determine the heritability of hormonal traits. Directional selection studies in the lab, as well as more recent field studies suggest that circulating corticosterone concentrations in birds have a heritable basis [96-100]. For free-living great tits, the degree of heritability of corticosterone levels has not yet been determined, but as a first step it can be tested whether corticosterone traits are consistent (or repeatable) within an individual. A trait is considered to be repeatable when in multiple (repeated) measures the within-individual variance is significantly smaller than the among-individual variance [1,101,102]. In other words, in repeatable traits there is less variance within an individual that has been sampled multiple times compared to the variance encountered in the population. Significant repeatability is often considered to be a measure for how effective selection may be acting on that trait, as well as an upper limit to heritability (but see [103]). Indeed, in captive great tits, variation within individuals in corticosterone traits after repeated capture-restraint protocols was smaller than among individuals, suggesting the existence of within-individual consistency [104,105]. Evidence for a more strictly defined statistical repeatability of baseline or stress-induced corticosterone concentrations in the literature is rather mixed (summarized in [56], see also [88,106,107]), although there is a trend for stress-induced corticosterone concentrations to be more repeatable than baseline levels.

To determine the repeatability of corticosterone concentrations for wild individuals we conducted studies in two European great tit populations (Southern Germany, the Netherlands [56,61]). In free-living great tits we only detected significant repeatability (r=0.26, p=0.025) in baseline corticosterone concentrations when samples were taken within a breeding context, i.e., when birds were sampled just prior to the breeding season in March and again during the actual parental phase in May [59]. This suggests that baseline corticosterone concentrations varied less within than among individuals at these two time points, such as when an individual that had relatively high corticosterone levels in March also had relatively high levels in May (although individuals tended to decrease baseline corticosterone levels from March to May [53]). As reviewed above, we found that individuals that displayed a specific directionality of seasonal plasticity (high March baseline corticosterone but low May levels) raised the most offspring. However, before being able to integrate the data on plasticity and reproductive success with those on repeatability, more work is required to determine the consistency of seasonal plasticity within individuals, its causation and its relationship with fitness. In contrast to the breeding phase, baseline corticosterone levels did not exhibit even a hint of repeatable variation when we sampled individuals across other seasons, even over relatively short time spans (days or weeks), or comparing samples from the parental phases (May) of two subsequent breeding seasons [56,61]. Stress-induced corticosterone levels were not repeatable either, that is to say within individual variance always exceeded among individual variance(although there was a trend for repeatability in one population, r=0.24, p=0.065 [61]).

How do we make sense of our data given the evidence from other bird species that corticosterone traits can be repeatable and likely have a heritable component (see above)? One possible explanation is that repeatability estimates may not always accurately reflect the degree of heritability of a trait, especially in highly plastic traits like circulating hormone concentrations [103,108]. This may especially be the case in wild populations that experience a wide variety of stimuli each day. By their nature, hormones are highly plastic traits, their concentrations changing readily in response to various environmental, social and internal stimuli [18]. Thus, it may not be too surprising that labile traits such as circulating corticosterone concentrations are not repeatable within free-living great tits. Such lability may be particularly strong for baseline corticosterone concentrations, which fluctuate with local conditions (ambient temperature, social circumstances) as well as with body condition or energy expenditure of an individual [40,54]. In contrast, stress-induced corticosterone concentrations likely indicate the capacity of an individual to hormonally respond to acute challenges and therefore may show a greater within-individual consistency. This interpretation is supported by the aforementioned study under more standardized conditions in captivity [105], in which great tits did show evidence for repeatability and the trend in the literature for repeatability to be significant for stress-induced corticosterone.

### Studying the evolution of plastic endocrine traits: reaction norm approaches as a way forward

**Figure 5:**
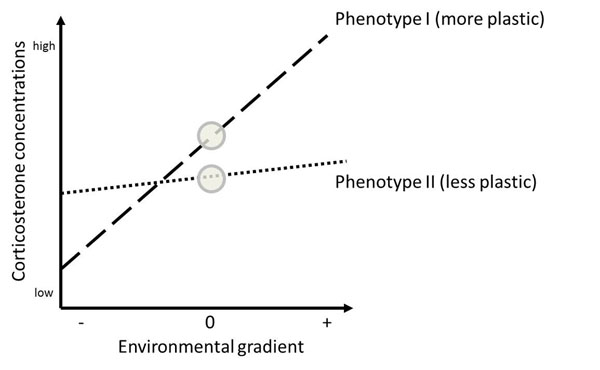
**Schematic representation of reaction norms for corticosterone.** Examples for two linear reaction norms, representing two different phenotypes (e.g., different individuals). Phenotype I shows a greater plasticity (steeper slope) in corticosterone concentrations along a given environmental gradient while phenotype II shows less plasticity (flatter slope). The two phenotypes also differ in elevation of their slopes, and in corticosterone concentrations in an average environment (when the gradient is 0; modified from [26], after [112]).

How can we make progress in understanding the evolution of hormonal traits given the issues above, particularly the prevalent plasticity in baseline corticosterone concentrations? One approach that has been valuable in studies of plastic physiological and behavioral traits is to quantify their reaction norms [20,109-114]. A reaction norm describes the phenotypic change in a trait along an environmental gradient (Fig. 5). Reaction norms thus allow the quantification of plastic responses of a trait to certain environmental changes, with the slope of the relationship indicating the degree and the direction of plasticity, and the intercept or elevation of the slope providing information about the overall value of the trait across the environmental gradient (or of the mean trait value if the environmental gradient is centered [20,26,109]). This approach will allow us to quantify the degree of change in hormone levels of great tits to given environmental, social or internal gradients [113]. Furthermore, repeatedly assessing the same individuals will allow us to determine the repeatability of hormonal reaction norms, for the same environmental gradient as well as across different contexts (see also section 4). Including information on the relatedness of tested individuals into statistical analyses will aid in estimating which aspects of the observed hormonal reaction norms have a heritable component. Establishing hormonal reaction norms for individuals will be challenging, as it will require repeatedly sampling the same individual, ideally using standardized variations in environmental or internal conditions. Experiments in captivity lend themselves more easily to such kinds of study, although field studies would be just as important.

The concept of reaction norms is beginning to be applied to the study of hormone-behavior relationships more generally, and a few examples for individual or population-level corticosterone reaction norms to salient environmental cues already exist [20,105,113,115-118]. There is strong evidence that reaction norms of physiological traits have a heritable basis and can evolve [114,119,120]. Determining reaction norms for endocrine traits therefore offers one highly promising approach for making progress in our understanding of the causes and functional significance of among-individual variation in hormone concentrations.

So far in this review, we have been focusing on hormones as a trait, assessed among-individual variation and considered evolutionary scenarios. In the following section we will shift perspectives and consider in more detail different types of quantitative relationships by which hormones can mediate behavioral traits. Although this is relevant for a range of behavioral traits that are regulated by different hormones, here we will focus on the role of testosterone in mediating behavior that is expressed during the breeding season.

## Within-individual plasticity: Enigmatic relationships between testosterone and behavior

**Figure 6:**
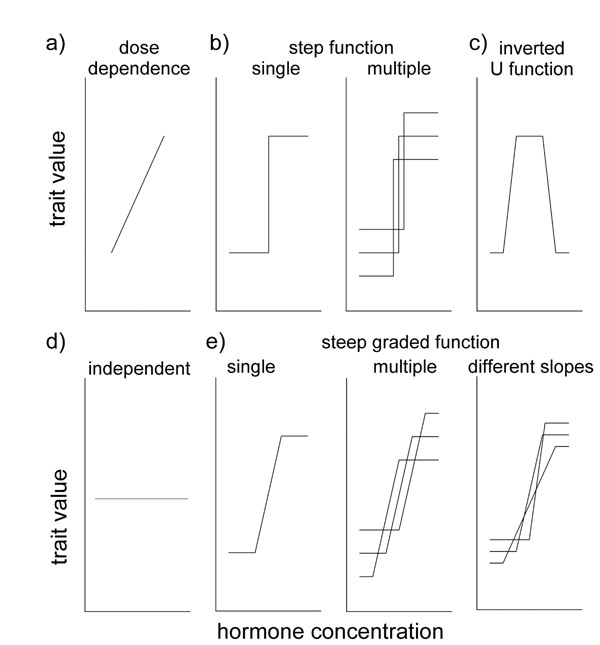
**Possible relationships between hormone concentrations and trait expression**. A) Dose-dependent relationship between hormone and trait; b) step function (on/off response); c) inverted U function; d) no relationship between hormone and trait; and e) steep graded function, i.e. a dose-dependent relationship within a narrow range of hormone concentration that may differ between individuals (Figure modified from [19,29,126]).

To improve our understanding of the role of hormones in behavioral flexibility we need to understand the exact ways in which hormones actually influence a trait. Many morphological traits that are regulated by testosterone show a linear relationship between testosterone concentrations (circulating or manipulated) and trait expression (Fig. 6a; e.g. [121-124]). For example, comb length in male red jungle fowl (*Gallus gallus*) is linearly related to circulating levels of plasma testosterone [124]. By contrast, it is increasingly becoming apparent that for hormone-behavior relationships such a graded relationship between hormone concentrations and behavioral expression seems to be the exception rather than the rule [19].

This puzzling observation prompted the idea that a step-function (Fig. 6b) rather than a linear function may more adequately describe the relationship between hormones and behavior. In this scenario, the behavior would be unlikely to be displayed if hormone concentrations remained below a certain threshold, but once a certain threshold was passed, the behavior would have the potential to be fully expressed, irrespective of any further increases in hormone concentrations [19,29] (Fig. 6b, single step function). Indeed, there is evidence for the existence of such step function relationships. For example, administration of a 2-5mm silastic capsule with exogenous testosterone to castrated male rats restored mating behavior to normal levels while the application of higher doses of testosterone was not more effective [125]. However, if individuals (for example the rats in the previous example) differed in their lower hormonal threshold necessary to elicit the behavior, this would – on a population level – generate the impression of a graded response similar to many morphological traits (Fig. 6b, multiple step function), thus explaining why this view is still prevalent in the literature.

Another possible relationship between the concentration of a hormone and the expression of a trait is an inverted U-function (Fig. 6c), where an elevation of hormone concentrations would first trigger an increase in the trait value, but after reaching a peak, further increases in hormone concentrations would lead to a decline in the trait value. An example for an inverted U-function includes the corticosterone-promoted perch hopping activity in white-crowned sparrows (*Zonotrichia leucophrys gambelii*), where medium amounts of exogenous corticosterone induced an increase in perch-hopping, while high amounts suppressed locomotor activity [37]. For completeness, the absence of a relationship between hormone and trait (flat line) is depicted in Fig. 6d.

Recently, Ball and Balthazart [126] questioned the existence of a step function, arguing that there was no *a priori* reason for behavior to be activated by hormones in a step-wise function, while other types of traits would show a graded response to hormones. Instead, these authors suggested the existence of a steep graded function (Fig. 6e), with a lower threshold of hormone concentrations, below which no behavior would be expressed, and a higher hormonal threshold, above which further increases in the hormone concentration would not elicit further increases in behavior [126]. But in between these two thresholds, the likelihood of behavioral expression would gradually increase in response to rising hormone concentrations (Fig. 6e). Evidence for such a steep graded response is provided by experiments in which increasing doses of exogenous testosterone induced a gradual increase in crowing behavior of quail [127]. Interestingly though, in this experiment the number of mounting attempts by male quail appeared to respond in a step-wise function to testosterone administration [127]. A steep graded response model can also be extended to predict behavioral responses to hormones on the population level. If the slopes of the graded responses differed among individuals, behaviors, and/or life-history stages (Fig. 6e, right), like with the step-wise model on a population level the results may give the impression of a linear relationship between hormone and behavior.

In our view, in contrast to the suggestion by Ball and Balthazart [126] there may indeed be good *a priori* reasons for why at least some behaviors may be regulated in a different way by hormones than morphological traits. Many morphological traits, such as wattles or combs, typically are traits that are continuously expressed (for example in a sex-dependent manner), but their degree of expression, such as their size or color, may vary. Hence, for such morphological traits, hormones could be expected to have a quasi-deterministic role in mediating their degree of expression, with a gradual increase in hormone concentrations leading to a gradual increase in trait expression. Behavior is different in that it is usually not expressed on a continuous basis, but only in specific and appropriate contexts (for example reproductive behavior). In this case, hormones would not be expected to deterministically regulate behavioral traits. Rather, their role would be to modulate the likelihood of a behavior to occur should the appropriate context arise. For example, given appropriate levels of circulating testosterone, a male may be more likely to show courtship behavior in the presence of sexually receptive females than when these females are absent or when his testosterone concentrations are low. Thus, hormones may be expected to have probabilistic effects on certain behaviors in facilitating or impeding their expression in specific contexts. Such probabilistic effects could very well be mediated in a simple step-wise manner, although it is also conceivable that a steep graded function could slowly increase the likelihood of the expression of the behavior in a dose-dependent manner once a threshold has been passed.

The nature of the relationship between a hormone and a behavioral trait could also differ between species, behaviors and also for different hormones. For example, the hormone testosterone lends itself well for mediating overall changes in reproductive state including in behavior, because it is produced in the testes and released at increased concentrations during the reproductive season. Once testosterone passes a certain threshold, this could result in the individual switching from a non-reproductive to a reproductive state, making reproductive behaviors such as courtship and aggression more likely to occur in the appropriate context [50]. On the other hand, for other hormones, such as corticosterone (or any other hormone involved in metabolism), gradual increases in its concentrations could be associated with gradual changes in the energetic state or allostatic load of an animal (e.g. [41]), which may very well lead to gradual changes in behavior, such as the ones observed in great tits feeding their offspring (see section 3). What are the implications of these different scenarios for the role of hormones in the regulation of behavioral phenotypic plasticity?

If a behavioral trait was mediated by a given hormone in a permissive step-wise manner (Fig. 6b), the role of this hormone for regulating behavioral flexibility within individuals would be limited. Instead, the hormone would primarily serve as an on/off-switch that either increases or decreases the likelihood of a behavior to occur in the appropriate context. The hormone would rather mediate a state, for example a reproductive versus a non-reproductive condition. In this case, plasticity might primarily be observed among individuals that differ in hormone thresholds, or within individuals when being in different life-history stages, ages, body conditions, etc. Thus, hormonal variation may be associated with behavioral plasticity only on a population level due to among-individual variation or within individuals only when they are sampled at different stages.

Hormones would have a much stronger role in mediating behavioral flexibility if they were associated with a behavior in a steep graded function relationship [126] (Fig. 6e). Behavioral flexibility would then mainly be mediated at hormone concentrations between the lower and the higher thresholds; within this range changes in hormone levels would directly translate to changes in the degree of behavioral expression. On a population level, individuals could differ both in their lower and higher thresholds, but also in the slope of the graded response in-between (Fig. 6e).

While such conceptual considerations regarding the nature of the relationship between hormones and behavior are important as outlined above, unfortunately the different models are often hard to distinguish in empirical studies. This issue is particularly relevant for population-level studies, where individuals are sampled only once. For example, recently Bonier et al. [54] conducted a comparative study to test whether concentrations of baseline glucocorticoids could be used to determine the severity of environmental challenges that individuals or populations experience, and whether higher concentrations of baseline corticosterone (indicating more challenging conditions) would relate to decreased Darwinian fitness (the ‘Cort-Fitness’ hypothesis). The data base Bonier et al. [54] analyzed was heterogeneous, including both observational and manipulative studies, and only about 50% of the published studies supported the prediction that glucocorticoid concentrations negatively relate to Darwinian fitness. In response to this finding, Dingemanse et al. [128] pointed out that the lack of support for the Cort-Fitness hypothesis may (at least partly) stem from individual differences in hormonal reaction norms, which reiterates the issues about the quantitative relationships between hormones and behavior discussed above [29,126] (Fig. 6). Thus, there is an emerging consensus in the field of behavioral endocrinology that estimates of individual reaction norms would help to disentangle questions related to how hormones regulate behavior, how behavior feeds-back on hormones, and how these processes relate to fitness. But how to practically address these questions – in particular in field studies – still represents a major challenge (see also [20]). Most field studies on birds (and probably most other vertebrates) suffer from the difficulty to repeatedly sample the same individual ([129], see also section 3). This limits the possibility to measure within-individual changes in testosterone, corticosterone or any other hormone that is associated with a change in environmental parameters, behavior, or other traits (but see [20] and [130] for promising approaches and results).

Identifying the quantitative relationship between concentrations of a particular hormone and its influence on the expression of a specific behavioral trait will help to elucidate phenotypic plasticity in a certain species. But even if that relationship was known for a given species, generalizing those findings to other species may prove difficult. It is becoming increasingly apparent that the degree to which certain types of behavior are under the influence of a hormone can vary quite drastically among species. Evidence that the hormonal control of behavior may be evolutionary quite flexible comes from studies of different species, which – upon superficial glance – appear to be ecologically quite similar. For example, if we compare the relationship between testosterone and territorial aggression among males of the four species black redstart (*Phoenicurus ochruros*), European robin (*Erithacus rubecula*), European stonechat (*Saxicola torquatus*), and western song sparrow (*Melospiza melodia morphna*), there seem to be substantial differences. All of these small, temperate zone songbird species are socially monogamous, establish territories both in the breeding and non-breeding seasons and show biparental care. Table 1 highlights some life-history characteristics as well as known relationships between testosterone and territorial aggression for each species (see also Table 1 for references). In sedentary western song sparrows, many components of male territorial aggression are promoted by testosterone, and experimentally blocking androgenic pathways is effective in reducing territorial aggression in both the breeding and non-breeding seasons. The song sparrow is also the only of the four species in which testosterone increases during male-male agonistic interactions in the breeding season, that is territorial behavior has a positive feed-back on hormone concentrations. In contrast to song sparrows, in migratory robins and in both migratory and non-migratory populations of stonechats some components of male territorial aggression are decreased by pharmacologically blocking androgenic pathways during the breeding season, but not if the same experimental manipulation is conducted outside of the breeding context. In migratory black redstarts, blocking androgenic pathways does not affect territorial aggression at all, neither during nor outside a breeding context. In the latter species, blocking androgen actions only affects structural parameters of male song that may be relevant for female choice (for references see Table 1), but not any type of aggressive territorial behavior. In stonechats, European robins and black redstarts, the display of territorial aggression also does not increase testosterone concentrations, i.e. a feedback of behavior on hormones is absent. The functional reasons for such inter-specific differences in the hormonal control of territorial behavior as well as the differences in the hormonal responsiveness to behavior are so far unexplained. Possibly, these differences in the involvement of testosterone in the modulation of territorial behavior (and vice versa) reflect evolutionary tinkering, i.e. there may be many ways in which evolutionary processes may link physiological mediators with behavioral outcomes. As a consequence, divergent mechanisms may have evolved for the hormonal regulation of similar types of behaviors such as territoriality, in particular if these behavioral traits have evolved independently in separate lineages.

**Table 1:** Life history parameters of 4 songbird species that may have independently evolved similar life-histories, but different control mechanisms for similar behavior (T= testosterone, breed= breeding, non-breed= non-breeding, R_male-male_= androgen responsiveness to male-male interactions).

	black redstart	European robin	European stonechat	western song sparrow
migration	yes	yes	yes/no	no
territoriality	breed/non-breed	breed/non-breed	breed/non-breed	breed/non-breed
occurrence of song	breed/non-breed	breed/(non-breed)	breed/(non-breed)	breed/non-breed
egg-laying period	3-4 months	3-4 months	3-4 months	3-4 months
R_male-male_	no	no	no*	yes
breeding aggression T-dependent	no	yes	yes	yes**
non-breed aggression T-dependent	no***	no	no	yes
linearity between behavior and T	no	no	no*	?

[55,134-154.]* Apfelbeck, Villavicencio, Flinks and Goymann, unpublished data;** as indicated by testosterone implants;***Goymann, Apfelbeck, Occasio and Windley, unpublished data.

## Conclusions

Animals live in environments that can fluctuate in a abiotic and biotic conditions over both short- and longer-term periods. Hormonally-mediated behavioral variation among and behavioral flexibility within individuals is important for adjusting behaviors to variation in environmental and social conditions. Since the rate of change in environmental conditions has increased in speed over past decades, necessitating appropriate changes in hormonally-mediated behaviors, the question of whether individuals and populations can adjust behaviors fast enough, either plastically or through evolutionary change in the underlying hormonal mechanisms, has become an urgent issue to address [131-133]. For example, for wild populations it is presently unclear whether individual plasticity can cope with environmental variation that has already or in the near future will exceed the range of conditions under which this plasticity has evolved, whether the presumed costs of plasticity may outweigh the benefits under altered environmental conditions, and whether the proximate processes that form the basis of plasticity can evolve rapidly enough to keep pace with changing environments [131-133]. Research on the relationships between hormones and behavior both among and within individuals will provide important contributions towards solving these questions.

## Declarations

Publication costs for this article were funded by the German Research Foundation (FOR 1232) and the Open Access Publication Fund of Bielefeld and Muenster University.

## Authors Contribution

MH and WG equally contributed in conceiving and writing the paper. Both authors read and approved the final manuscript.

## Competing interests

The authors declare that they have no competing interests.
